# Supplementary material for: Sequencing whole genomes of the West Javanese population in Indonesia reveals novel variants and improves imputation accuracy
Source: Front Genet. 2025 Feb 7;15:1492602. doi: 10.3389/fgene.2024.1492602 (PMC11843580; doi:10.3389/fgene.2024.1492602)
Supplement: Supplementary file 1 [file DataSheet1.zip › Supplementary Table 1.pdf]

Supplementary Table 1 Number of rare (MAF < 0.01) Single Nucleotide Variants (SNVs) and Insertions/Deletions (InDels) in the West Javanese Whole Genome Sequencing dataset. The number and percentage of variants detected by WGS are indicated.

chr	SNV			InDel		
	n	MAF<0.01 (%)	Novel (%)	n	MAF<0.01 (%)	Novel (%)
1	1,142,385	542,341 (47.47)	159,050 (13.92)	203,229	77,109 (37.94)	91,251 (44.90)
2	1,125,085	493,890 (43.90)	131,482 (11.69)	217,566	86,852 (39.92)	96,341 (44.28)
3	998,139	447,438 (44.83)	125,241 (12.55)	175,037	68,860 (39.34)	77,211 (44.11)
4	1,029,070	463,698 (45.06)	132,752 (12.90)	171,690	66,233 (38.58)	73,229 (42.65)
5	912,383	418,083 (45.82)	121,971 (13.37)	156,121	62,126 (39.79)	68,222 (43.70)
6	923,747	442,157 (47.87)	132,683 (14.36)	149,907	57,031 (38.04)	64,322 (42.91)
7	811,295	359,619 (44.33)	103,287 (12.73)	148,801	58,230 (39.13)	65,590 (44.08)
8	832,534	407,651 (48.97)	124,267 (14.93)	124,224	49,907 (40.18)	54,998 (44.27)
9	641,048	298,000 (46.49)	88,948 (13.88)	101,379	38,170 (37.65)	44,857 (44.25)
10	713,173	322,913 (45.28)	88,164 (12.36)	118,280	44,339 (37.49)	52,154 (44.09)
11	697,223	324,751 (46.58)	90,386 (12.96)	113,123	43,917 (38.82)	49,546 (43.80)
12	667,314	298,554 (44.74)	79,976 (11.98)	123,633	47,061 (38.07)	55,351 (44.77)
13	528,819	249,104 (47.11)	71,526 (13.53)	89,867	34,850 (38.78)	38,011 (42.30)
14	479,730	225,262 (46.96)	64,511 (13.45)	82,810	31,675 (38.25)	36,192 (43.70)
15	459,534	227,984 (49.61)	68,859 (14.98)	75,693	29,030 (38.35)	33,711 (44.54)
16	446,985	211,452 (47.31)	57,336 (12.83)	69,332	25,029 (36.10)	32,502 (46.88)
17	411,226	202,703 (49.29)	58,750 (14.29)	78,585	29,261 (37.23)	37,932 (48.27)
18	404,206	185,227 (45.82)	53,341 (13.20)	64,926	24,300 (37.43)	27,625 (42.55)
19	337,343	155,476 (46.09)	41,244 (12.23)	63,924	21,385 (33.45)	30,532 (47.76)
20	315,433	149,288 (47.33)	43,024 (13.64)	53,219	20,202 (37.96)	24,049 (45.19)
21	203,343	94,607 (46.53)	26,070 (12.82)	33,382	12,999 (38.94)	13,629 (40.83)
22	203,143	96,216 (47.36)	26,810 (13.20)	34,882	12,958 (37.15)	15,737 (45.11)
**Total**	**14,283,158**	**6,616,414 (46.32)**	**1,889,678 (13.23)**	**2,449,610**	**941,524 (38.44)**	**1,082,992 (44.21)**
