# Supplementary material for: Sequencing whole genomes of the West Javanese population in Indonesia reveals novel variants and improves imputation accuracy
Source: Front Genet. 2025 Feb 7;15:1492602. doi: 10.3389/fgene.2024.1492602 (PMC11843580; doi:10.3389/fgene.2024.1492602)
Supplement: Supplementary file 2 [file DataSheet2.docx]

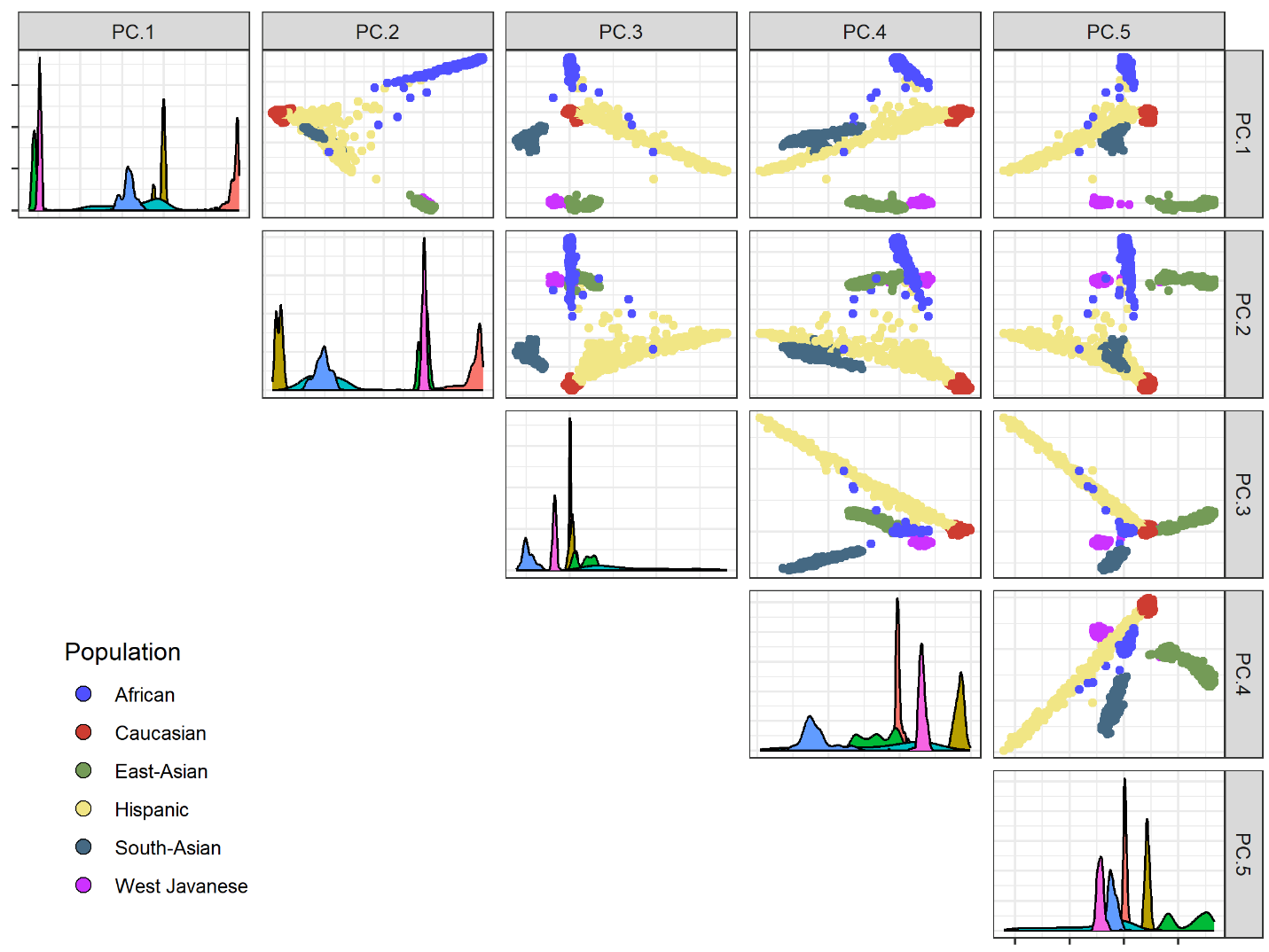


**Supplementary Figure 1** The first five principal components showing the genetic positioning of the West Javanese population in relation to the 1000 Genome population


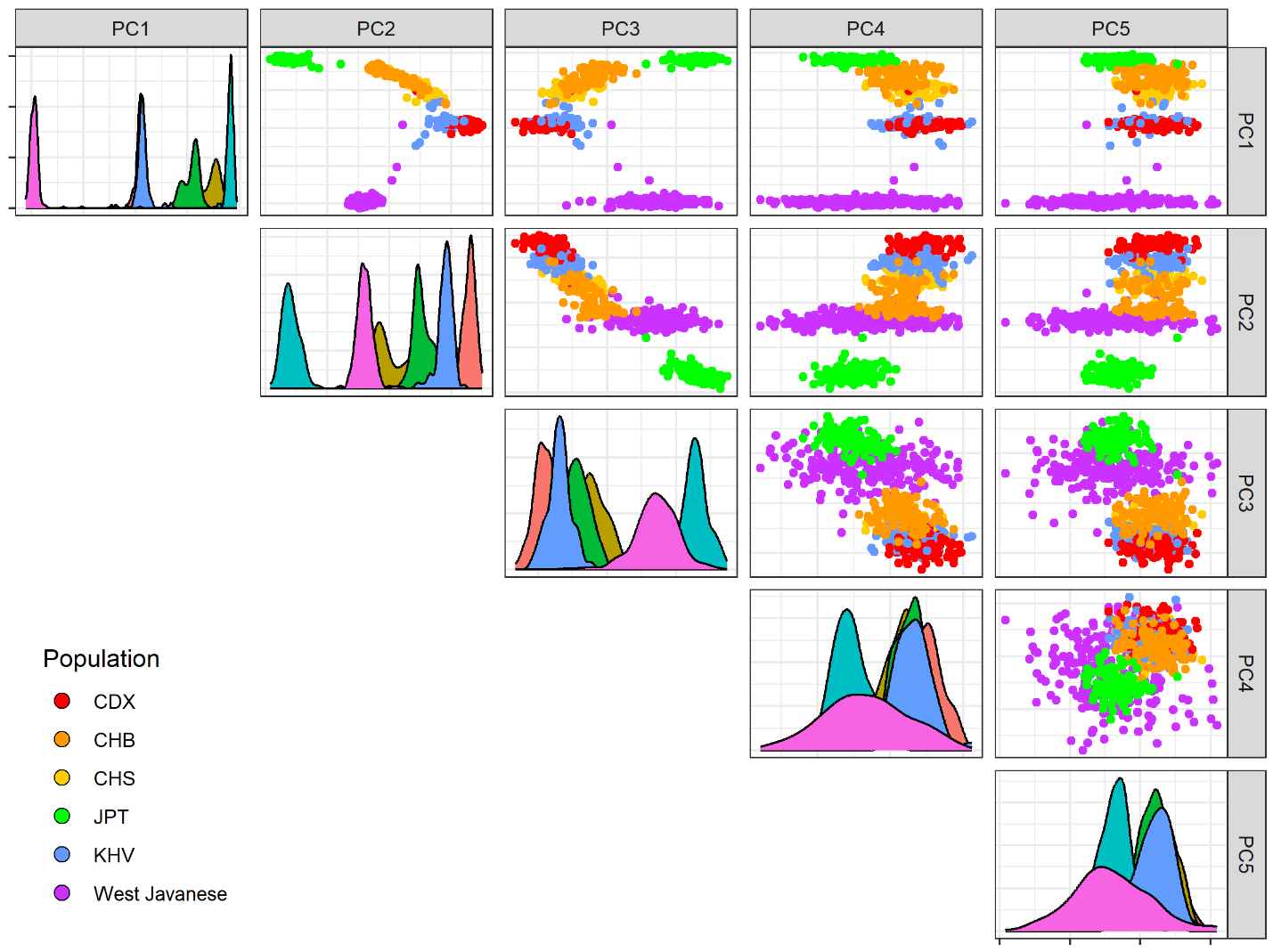


**Supplementary Figure 2** The first five principal components showing the genetic positioning of the West Javanese population in relation to other ethnicities within the 1000 Genome East-Asian population.


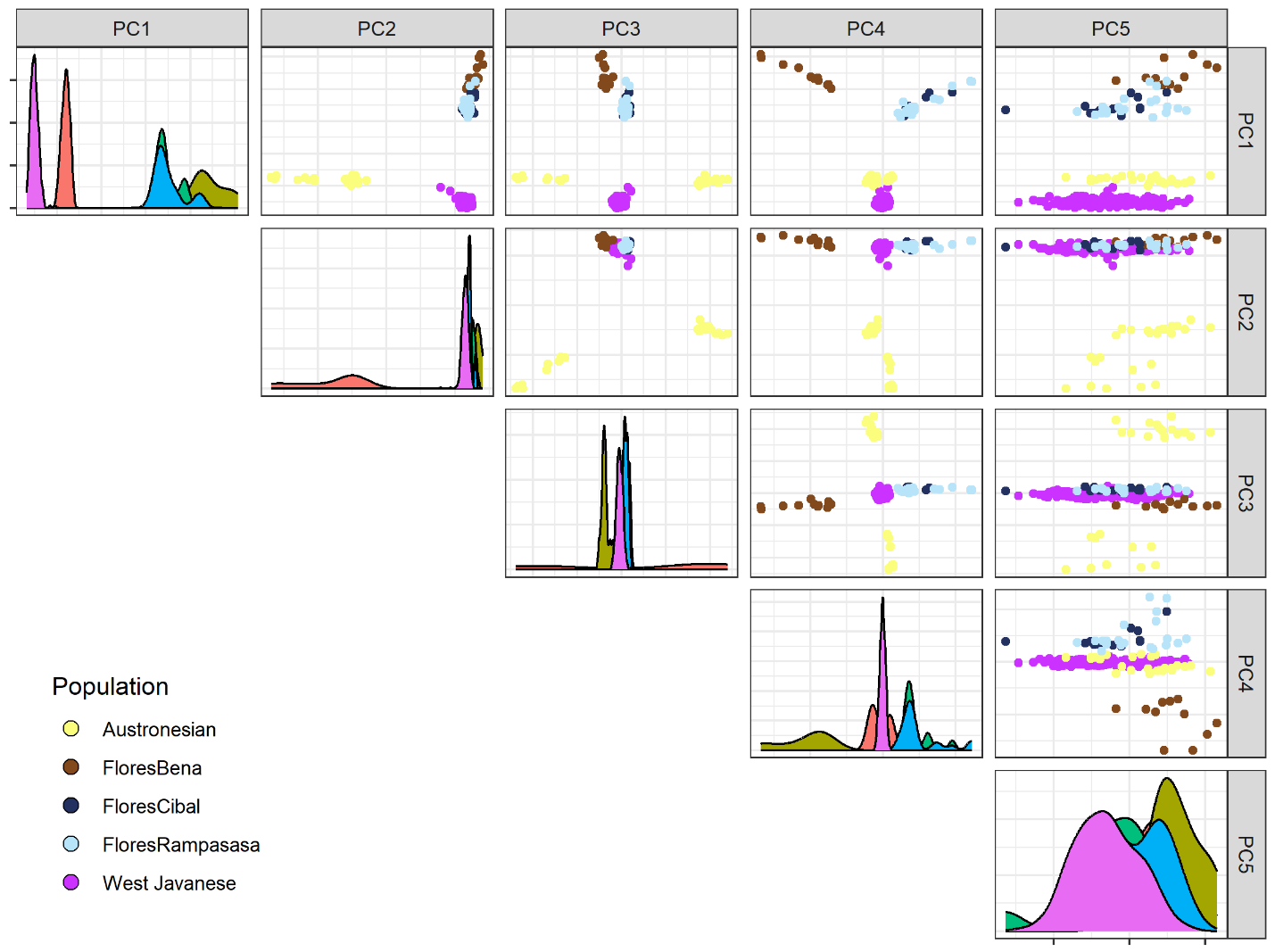


**Supplementary Figure 3** The first five principal components showing the genetic positioning of the Indonesian population (Flores) obtained from the *GenomeAsia* 100K project.
